# Assessment of Biochemical Parameters of the Oral Fluid before and after Using Office Teeth Whitening Systems

**DOI:** 10.3390/dj10100178

**Published:** 2022-09-21

**Authors:** Elena A. Ryskina, Frida N. Gilmiyarova, Oksana A. Magsumova, Mikhail A. Postnikov, Tatiana A. Lobaeva, Dmitry D. Zhdanov

**Affiliations:** 1 Shemyakin-Ovchinnikov Institute of Bioorganic Chemistry, Faculty of Biology and Biotechnology, Higher School of Economics (HSE University), 20 Myasnitskaya St., 117418 Moscow, Russia; 2 Department of Therapeutic Dentistry, Samara State Medical University, Chapaevskaya St., 89, 443099 Samara, Russia; 3 Department of Biochemistry, Peoples’ Friendship University of Russia, 8 Miklukho-Maklaya St., 117198 Moscow, Russia; 4 Laboratory of Medical Biotechnology, Institute of Biomedical Chemistry, 10/8 Pogodinskaya St., 119121 Moscow, Russia

**Keywords:** oral fluid, indicators of mineral metabolism, alkaline phosphatase, superoxide dismutase, in-office teeth whitening systems

## Abstract

One of the most important functions of the oral fluid is to maintain oral homeostasis. In-office teeth whitening systems are able to change the mineral metabolism and the activity of a number of enzymes in the oral fluid, but there are conflicting data in publications about this. The aim of this study was to compare the effect of Opalescense Boost, ZOOM Advance POWER, and ZOOM Phillips White Speed, which contain different percentages of hydrogen peroxide, on the performance of oral fluid. After the procedure of whitening teeth with the studied in-office systems, the concentration of calcium in the oral fluid increased, and the activity of alkaline phosphatase decreased. Calcium levels returned to baseline values after 30 days, and alkaline phosphatase activity returned after 14 days. There was no significant difference in the changes in calcium concentration and alkaline phosphatase activity between different tooth whitening systems. Chemical teeth whitening with the Opalescense Boost system caused the largest change in the activity of superoxide dismutase in the oral fluid compared to the ZOOM Advance POWER and ZOOM Phillips White Speed photocatalytic teeth whitening systems. An increase in the activity of superoxide dismutase by +75.5% was shown immediately after the procedure of teeth whitening with the Opalescense Boost system, which indicated an increase in the power of antioxidant defense mechanisms. To assess the effectiveness and safety of using various whitening systems, it is possible to study the dynamics of the activity of superoxide dismutase, which reflects the processes of antioxidant protection of the oral cavity.

## 1. Introduction

Oral fluid or mixed saliva is a secretion product of the salivary glands in the composition of epithelial cells, leukocytes, microorganisms, micro and gingival fluid. Saliva itself is a complex of fluids produced by specific glands and relies on a system of ducts into the oral cavity. This biological fluid has a role in diagnosing and monitoring the patient’s health. Oral fluid plays an important role in maintaining the homeostasis of the oral cavity: it takes part in the mineralization of the hard tissues of the tooth, exhibits antioxidant functions, reduces the activity of microorganisms in the oral cavity, and participates in moisturizing the oral mucosa. Almost all the chemical elements of the periodic table are found in human saliva, but calcium and phosphorus are of paramount importance, since they maintain the optimal composition of the components of dental tissue.

Oral fluid is the least studied and most underestimated biological environment in the human body which may have a diagnostic significance. According to the results of numerous studies, peroxide compounds that make up teeth whitening gels can affect not only the chemical composition of enamel but also the indicators of mineral metabolism and the activity of enzymes in the oral fluid [1,2,3,4], but in publications, there are no data on the effect of in-office teeth whitening systems on oral fluid homeostasis. 

The procedure of teeth whitening is carried rather often due to the great interest of patients in this type of procedure. It brings very good results taking into account the aesthetic aspects. However, teeth whitening also causes great concern to patients and dentists due to its potential harmful effects on tooth tissue and the biochemical environment in the mouth cavity [5,6]. The purpose of this study was to compare the effect of different in-office teeth whitening systems such as Opalescense Boost, ZOOM Advance POWER, and ZOOM Phillips White Speed, which contain different percentages of hydrogen peroxide, on the biochemical composition of the oral fluid

## 2. Materials and Methods

### 2.1. Patients and Whitening Systems

This study was conducted at the Department of Therapeutic Dentistry of Samara State Medical University. The study was approved by the ethics committee of Samara State Medical University (protocol 213, 23-04-2020). This protocol was compliant with the Helsinki Declaration of 1975, as revised in 2000. The protocol was explained to all participants, and after answering every question, they signed the informed consent form before their saliva samples were taken for analysis. The study involved 32 patients aged 18 to 32 years. The inclusion criteria were the following: enamel pigmentation; aesthetic preferences of a patient; tetracycline-stained teeth; the dashed, spotty, chalky-mottled form of generalized fluorosis of the teeth; age-related changes of enamel color; and local enamel hypoplasia of mild severity. The exclusion criteria were the following: childhood and adolescence; enlarged dental pulp chamber; periodontal tissue diseases; the presence of orthopedic and/or orthodontic appliances; all types and stages of caries; hyperesthesia; the presence of tumors, bronchial asthma, and uncontrolled increase of blood pressure. 

Before the start of the whitening procedure, the oral cavity was sanitized. Three systems of teeth whitening were used—chemical whitening with the Opalescense Boost system (Ultradent Products Inc. South Jordan, UT, USA), light catalytic whitening ZOOM Advance POWER (ZOOM 3) (Philips, Eindhoven, The Netherlands), and light catalytic whitening ZOOM Phillips White Speed (ZOOM 4) (Philips, Eindhoven, The Netherlands). Chemical whitening with the Opalescense Boost system was carried out on the basis of a gel with 40% hydrogen peroxide content. The teeth whitening system ZOOM Advance POWER included a gel based on 25% hydrogen peroxide activated by a halogen lamp emitting light in the range of 350–400 nm. Teeth whitening with the ZOOM Phillips White Speed (ZOOM 4) system was also carried out on the basis of a gel with 25% hydrogen peroxide, which was activated by an LED lamp operating in the range of 400–505 nm. Chemical teeth whitening was performed using the Opalescence Boost 40% (Ultradent) system, based on 40% hydrogen peroxide. The whitening gel was applied to the surface of the teeth three times, the exposure was 20 min.

In catalytic bleaching, teeth whitening using systems ZOOM Advance Power and Zoom Phillips White Speed, based on which a 25% hydrogen peroxide whitening gel was applied to the surface of the teeth three times, the exposure time was 15 min. All patients were divided into 3 groups depending on the use of the whitening system. Patients were allowed to choose the bleaching system based on their personal preferences as to date there is no difference in the criteria of their use. Teeth whitening with the Opalescense Boost system was performed in the first group of patients (8 people), teeth whitening with the ZOOM Advance POWER system was performed in the second group of patients (8 people), and teeth whitening with the ZOOM Phillips White Speed system was performed in the third group of patients (16 people). The ZOOM 4 whitening system is the most popular today. The main difference between ZOOM 4 and ZOOM 3 is that it is considered to have a gentler effect on the teeth. A safer whitening by the ZOOM 4 system was a key factor why it was chosen by 50% of the study group.

### 2.2. Collection of Saliva and Measurement of Its Biochemical Characteristics

Oral fluid samples were taken before the tooth whitening procedure, immediately after, after 2 weeks, and after a month. Before sampling the oral fluid, all patients rinsed their mouths with drinking water. Samples were collected 30 min after a meal. Collection of oral fluid was carried out in a sterile test tube, followed by freezing at a temperature of −20 °C. The next step was defrosting the test tubes for 10 h at a temperature of +3 °C; after defrosting, the temperature of the samples was brought to room temperature. Afterwards, the oral fluid was aliquoted into Eppendorf tubes and centrifuged at 5000× *g* for 5 min in an Eppendorf MiniSpin centrifuge.

The determination of indicators of the oral fluid was carried out on an Aquarius CE 7200 spectrophotometer (Cecil Instruments, Cambridge, UK). The concentrations of calcium, phosphorus, magnesium, and iron and the activities of acid phosphatase (EC 3.1.3.2), alkaline phosphatase (EC 3.1.3.1), and superoxide dismutase (EC 1.15.1.1) were determined. The calcium concentration was determined by the photometric method with o-cresolphthalein complexone [7], the phosphorus content was measured by the molybdate UV method [8], the magnesium concentration was measured by the xylidyl blue method [9], and the iron concentration was determined by the method with chromazurol B [10] using ready-made Human kits (Human, Wiesbaden, Germany). Serum Humatrol (Human, Wiesbaden, Germany) was used as a control material.

The determination of the activity of phosphatases in the oral fluid was carried out using ready-made kits from Human (Human, Wiesbaden, Germany). The determination of the activity of alkaline phosphatase was carried out by an optimized standard method according to the recommendation of the German Association for Clinical Chemistry with p-nitrophenyl phosphate [11] and in the case of acid phosphatase by the method with α-naphthyl phosphate [12]. Superoxide dismutase activity was determined spectrophotometrically based on the ability of the enzyme to inhibit the autooxidation of adrenaline in an alkaline medium. The reaction rate was evaluated by the value of the optical density of the accumulating product of autooxidation of adrenaline, which has an absorption maximum at a wavelength of 347 nm, formed in the absence and presence of an aliquot of oral fluid. The calculation was carried out in arbitrary units of enzyme activity according to the formula: (1 − (ΔDtest/ΔDcontrol)) × 100%, where ΔDtest is the extinction in the test sample and ΔDcontrol is the extinction in the control sample. One arbitrary unit was taken as 1% inhibition of enzyme activity.

### 2.3. Statistical Analysis

The statistical data analysis was performed using SPSS 25 software (IBM SPSS Statistics, Armonk, NY, USA). Descriptive statistics used M—mean value, m—standard error of the mean, and Δ%—difference in percentage of sample mean values (Mtest/Mcontrol) × 100%).

Comparisons of independent groups were performed using a Kruskal–Wallis analysis (Pk–W) followed by pairwise comparisons using the Mann–Whitney test. Differences were considered statistically significant at values *p* < 0.05.

## 3. Results

The study of the effect of tooth whitening systems showed that the concentration of some indicators of mineral metabolism and the activity of enzymes changed in the oral fluid. The concentration of calcium in the oral fluid is less than that in the blood serum and is normally 0.75–3.00 mM [13]. In the three groups of patients, there was a statistically significant increase in the concentration of calcium in the oral fluid after the tooth whitening procedure and 14 days after whitening (Table 1).

Calcium levels returned to baseline values after 30 days. There was no significant difference in the changes in calcium concentration after the tooth whitening procedure between the patient groups.

The phosphorus content in the oral fluid reached 2.2–6.5 mM. In the third group of patients, the phosphorus concentration before the tooth whitening procedure was 4.31 ± 0.13 mM. There was a decrease in the concentration of phosphorus immediately after the tooth whitening procedure to 3.69 ± 0.22 (*p* = 0.026), and the phosphorus concentration returned to the initial value after 14 days. The ratio of calcium/phosphorus (Ca/P) in the oral fluid increased immediately after the tooth whitening procedure and returned to baseline values after 14 days, reflecting the change in the content of calcium and phosphorus in the oral fluid.

The activities of alkaline phosphatase (EU/L), acid phosphatase (EU/L), and superoxide dismutase in the oral fluid were determined at different periods of observation. The activity of alkaline phosphatase significantly decreased after the whitening procedure in all three groups of patients and returned to baseline values after 14 days (Table 2).

Acid phosphatase activity (EU/L) remained unchanged in the three groups of patients. For Opalescense Boost, the indicator value before whitening was 0.47 ± 0.09 EU/L, and 30 days after whitening, it was 0.47 ± 0.06 EU/L. For ZOOM Advance POWER (ZOOM 3), the initial value was 0.50 ± 0.09 EU/L, while 30 days after whitening, the value was 0.50 ± 0.08 EU/L. For ZOOM Phillips White Speed (ZOOM 4), the baseline value before whitening was 0.48 ± 0.06 EU/L and the final value after 30 days was 0.48 ± 0.05 EU/L.

In the control (without oral fluid), a gradual increase in extinction was noted as a consequence of the formation of adrenochrome in the process of adrenaline autooxidation. In experimental tests (with oral fluid), the increase in extinction slowed down as a consequence of the inhibition of adrenaline autooxidation under the action of superoxide dismutase. The SOD activity in the oral fluid in the three study groups increased immediately after the tooth whitening procedure; after 14 days, a significant change in SOD activity was preserved. The enzyme activity returned to its initial values after 30 days (Table 3).

## 4. Discussion

During tooth whitening, hydrogen peroxide diffuses into the tooth enamel and dissociates to form free radicals, such as hydroxyl radicals, perhydroxyl radicals, perhydroxyl anions, and superoxide anions, which attack organic pigmented molecules [14]. Some studies show that after teeth whitening, a significant decrease in the concentration of certain elements in the enamel of the teeth is determined [15,16]. Other studies report no changes in tooth enamel when exposed to hydrogen peroxide [17,18].

All in-office tooth whitening systems tested produced changes in various parameters of the oral fluid. The results obtained indicate an increase in the concentration of calcium in the oral fluid, which occurs as a result of the removal of ions from the enamel of the teeth. The concentration of phosphorus in the oral fluid did not change after the tooth whitening procedure. The calcium/phosphorus ratio is an important indicator of the process of remineralization of dental hard tissues. The results obtained show a change in the Ca/P ratio after the tooth whitening procedure toward an increase due to an increase in the concentration of calcium in the oral fluid. The Ca/P ratio returned to the initial values after a month, which is a favorable prognostic sign of tooth remineralization. According to Dourado P. et al., the use of teeth whitening systems with hydrogen peroxide leads to the leaching of calcium and phosphorus from the enamel of the teeth [19].

There was a tendency toward an increase in the concentration of magnesium in the oral fluid in all groups of patients immediately after the tooth whitening procedure; the concentration of iron after tooth whitening in the study groups did not change at different periods of observation. In studies by Cakir F.Y. et al., it was shown that magnesium was one of the first elements removed during the reaction of peroxide from the tooth surface [20]. The formation of reactive oxygen species can stimulate an increase in the magnesium content in the oral fluid for the element to fulfill its antioxidant function, i.e., the reduction of peroxide radicals, which is consistent with the data of Inonu E. et al. [21].

Acid and alkaline phosphatases function to maintain the level of phosphorus in saliva. The activity of phosphatases in the oral fluid increases, as a rule, in inflammatory processes of the oral cavity. In the research of Sanches, it was shown that professional teeth whitening increased the activity of alkaline phosphatase, lactate dehydrogenase, aspartate, and alanine aminotransferases in the oral fluid, thereby affecting the homeostasis of the oral cavity by reducing the remineralizing potential of the oral fluid [22]. Soares D.G. et al. found that the activity of alkaline phosphatase in the oral fluid decreased when dental tissue was exposed to hydrogen peroxide, which is a response to oxidative stress [23,24].

The study of the activity of alkaline phosphatase in the oral fluid after the tooth whitening procedure showed that the activity of alkaline phosphatase decreased slightly in the three groups of patients immediately after using the tooth whitening procedure and recovered after 14 days. No intergroup differences were found.

The system of antioxidant protection of the oral cavity includes superoxide dismutase. The mechanism of tooth whitening is based on the ability of hydrogen peroxide to generate free radicals, which causes an increase in the activity of superoxide dismutase in the oral fluid after the tooth whitening procedure. There was a significant increase in SOD activity after tooth whitening and a gradual return to baseline values after 30 days. The SOD activity in the oral fluid immediately after the bleaching procedure and 14 days after the bleaching procedure was the highest in the first group of patients. This whitening system uses a gel with 40% hydrogen peroxide content, and the lowest in the third group of patients. This whitening system uses gel with a 25% hydrogen peroxide content. Comparisons of independent groups using a Kruskal–Wallis one-way analysis of variance showed significant differences between groups 1 and 2 (*p* = 0.036) and between groups 1 and 3 (*p* = 0.02). The Opalescense Boost teeth whitening system caused an increase in superoxide dismutase activity by +75.5%, which indicated an increase in the power of antioxidant defense mechanisms. It was shown that chemical teeth whitening with the Opalescense Boost system had the greatest effect on SOD activity in comparison with the ZOOM Advance POWER and ZOOM Phillips White Speed systems.

There was no significant difference in oral fluid indicators when using various bleaching systems, with the exception of superoxide dismutase activity. To assess the effectiveness and safety of various whitening systems, it is possible to study the dynamics of the activity of superoxide dismutase, which reflects the processes of antioxidant protection of the oral cavity.

The results obtained confirmed that teeth whitening procedures lead to calcium leaching from tooth enamel; however, these changes are reversible and can be eliminated by the use of remineralizing toothpastes.

## 5. Conclusions

All teeth whitening systems cause an increase in the concentration of calcium in the oral fluid and a gradual return to the initial values after 30 days. The activity of alkaline phosphatase decreased in patients after using the teeth whitening procedure and recovered after 14 days in three study groups. There was a significant increase in SOD activity after teeth whitening and a gradual return to baseline values after 30 days for all teeth whitening systems used. 

There was no significant difference in oral fluid parameters when using different bleaching systems, with the exception of the activity of superoxide dismutase. The Opalescense Boost teeth whitening system caused an increase in superoxide dis-mutase activity by +75.5%, which indicated an increase in the power of antioxidant defense mechanisms.

## Figures and Tables

**Table 1:** Concentrations of calcium in the oral fluid before and at different times after tooth whitening (mM).

Patient Groups	Saliva Samples			
	Before Whitening	Right after Whitening	14 Days after Whitening	30 Days after Whitening
1st group	2.11 ± 0.15	3.39 ± 0.28 Δ% + 60.0	2.44 ± 0.11 Δ% + 15.6	2.20 ± 0.30 Δ% + 4.3
		*p* < 0.05	*p* < 0.05	0.327
2nd group	2.16 ± 0.14	3.15 ± 0.15 Δ% + 45	2.39 ± 0.08 Δ% + 10.0	2.17 ± 0.12 Δ% + 0.5
		*p* < 0.05	*p* < 0.05	0.123
3rd group	2.16 ± 0.06	3.32 ± 0.11 Δ% + 53	2.40 ± 0.05 Δ%11.1	2.20 ± 0.08 Δ%1.9
		*p* < 0.001	*p* < 0.01	0.170

M—mean value, m—standard error of the mean, Δ%—difference in percentage of sample mean values (Mtest/Mcontrol) × 100% (“+” indicates the increase in % relative to the value in the “before whitening” group; “−“ indicates the decrease in % relative to the value in the “before whitening” group).

**Table 2:** The activity of alkaline phosphatase in the oral fluid before and at different times after tooth whitening (EU/L).

Patient Groups	Saliva Samples			
	Before Whitening	Right after Whitening	14 Days after Whitening	30 Days after Whitening
1st group	21.02 ± 1.03	17.92 ± 0.74 Δ% − 14.7	19.99 ± 0.45 Δ% − 5.1	21.71 ± 1.10 Δ% + 3.3
	-	*p* < 0.05	0.140	0.102
2nd group	21.71 ± 0.62	19.30 ± 0.74 Δ% − 11.1	20.33 ± 1.37 Δ% − 6.4	21.37 ± 0.69 Δ% − 1.7
		*p* < 0.05	0.595	0.680
3rd group	22.06 ± 0.62	19.13 ± 0.77 Δ% − 13.3	20.68 ± 0.80 Δ% − 6.3	21.88 ± 0.89 Δ% − 0.8
		*p* < 0.01	0.133	0.318

M—mean value, m—standard error of the mean, Δ%—difference in percentage of sample mean values (Mtest/Mcontrol) × 100% (“+” indicates the increase in % relative to the value in the “before whitening” group; “-“ indicates the decrease in % relative to the value in the “before whitening” group).

**Table 3:** Activity of superoxide dismutase (SOD) in the oral fluid before and at different times after tooth whitening (conventional units).

Patient Groups	Saliva Samples			
	Before Whitening	Right after Whitening	14 Days after Whitening	30 Days after Whitening
1st group	32.4 ± 2.7	56.9 ± 2.7 Δ% + 75.6	50.1 ± 2.2 Δ% + 54.5	34.8 ± 6.1 Δ% + 7.4
		*p* < 0.05	*p* < 0.05	*p* = 0.075
2nd group	33.6 ± 2.8	57.9 ± 2.1 Δ% + 72.3	51.1 ± 4.0 Δ% + 52.1	37.1 ± 3.6 Δ% + 10.4
		*p* < 0.05	*p* < 0.05	*p* = 0.055
3rd group	32.4 ± 1.1	54.9 ± 1.5 Δ% + 69.4	45.1 ± 2.7 Δ% + 39.2	34.7 ± 1.6 Δ% + 7.1
		*p* < 0.001	*p* < 0.01	*p* = 0.070

M—mean value, m—standard error of the mean, Δ%—difference in percentage of sample mean values (Mtest/Mcontrol) × 100% (“+” indicates the increase in % relative to the value in the “before whitening” group; “-“ indicates the decrease in % relative to the value in the “before whitening” group).

## Data Availability

The data presented in this study are available on request from the corresponding author. The data are not publicly available due to ethical restrictions.

## References

[1] Vilhena K.F.B., Nogueira B.C.L., Fagundes N.C.F., Loretto S.C., Angelica R.S., Lima R.R., Silva E Souza M.H.J. (2019). Dental enamel bleached for a prolonged and excessive time: Morphological changes. PLoS ONE.

[2] Magsumova O.A., Ryskina E.A., Postnikov M.A., Tkach T.M., Polkanova V.A. (2020). Change in the sensitivity of hard tissues of teeth after the procedure of office teeth whitening. Inst. Dent..

[3] Godinho J., Silveira J., Mata A., Carvalho M.L., Pessanha S. (2014). Effect of bleaching gel in Ca, P and Zn content in tooth enamel evaluated by μ-EDXRF. Nucl. Instrum. Methods Phys. Res. Sect. B Beam Interact. Mater. Atoms.

[4] Cakir F.Y., Ergin E., Gurgan S., Sabuncuoglu S., Arpa C.S., Tokgoz İ., Ozgunes H., Kiremitci A. (2015). Effect of bleaching on mercury release from amalgam fillings and antioxidant enzyme activities: A pilot study. J. Esthet. Restor. Dent. Off. Publ. Am. Acad. Esthet. Dent..

[5] Alkahtani R., Stone S., German M., Waterhouse P. (2020). A review on dental whitening. J. Dent..

[6] Panizzi C., Chiesa A., Preda C., Lanteri V., Scribante A., Butera A., Segù M. (2021). Evaluation of a post oral hygiene carbamide peroxide bleaching gel: A pilot study. Int. J. Clin. Dent..

[7] Barnett R.N., Skodon S.B., Goldberg M.H. (1973). Performance of “kits” used for clinical chemical analysis of calcium in serum. Am. J. Clin. Pathol..

[8] Gamst O., Try K. (1980). Determination of serum-phosphate without deproteinization by ultraviolet spectrophotometry of the phosphomolybdic acid complex. Scand J. Clin. Lab Investig..

[9] Bohuon C. (1962). Mircodetermination of magnesium in various biological media. Clin. Chim. Acta.

[10] Garcic A. (1979). A highly sensitive, simple determination of serum iron using chromazurol B. Clin. Chim. Acta.

[11] Schlebusch H., Rick W., Lang H., Knedel M. (1974). Standards in the activities of clinically important enzymes. Dtsch. Med. Wochenschr..

[12] Hillmann G. (1971). Continuous photometric measurement of prostate acid phosphatase activity. Z. Klin. Chem. Klin. Biochem..

[13] Carey C.M., Vogel G.L. (2000). Measurement of Calcium Activity in Oral Fluids by Ion Selective Electrode: Method Evaluation and Simplified Calculation of Ion Activity Products. J. Res. Natl. Inst. Stand. Technol..

[14] Minoux M., Serfaty R. (2008). Vital tooth bleaching: Biologic adverse effects-a review. Quintessence Int..

[15] Joiner A. (2006). The bleaching of teeth: A review of the literature. J. Dent..

[16] Souza R.O.A., Lombardo G.H.L., Pereira S.M.B., Zamboni S.C., Valera M.C., Araujo M.A.M., Ozcan M. (2010). Analysis of tooth enamel after excessive bleaching: A study using scanning electron microscopy and energy dispersive x-ray spectroscopy. Int. J. Prosthodont..

[17] Llena C., Esteve I., Forner L. (2018). Effects of in-office bleaching on human enamel and dentin. Morphological and mineral changes. Ann. Anat.-Anat. Anz..

[18] Mielczarek A., Klukowska M., Ganowicz M., Kwiatkowska A., Kwaśny M. (2008). The effect of strip, tray and office peroxide bleaching systems on enamel surfaces in vitro. Dent. Mater..

[19] Dourado Pinto A.V., Carlos N.R., do Amaral F.L.B., França F.M.G., Turssi C.P., Basting R.T. (2019). At-home, in-office and combined dental bleaching techniques using hydrogen peroxide: Randomized clinical trial evaluation of effectiveness, clinical parameters and enamel mineral content. Am. J. Dent..

[20] Cakir F.Y., Korkmaz Y., Firat E., Oztas S.S., Gurgan S. (2011). Chemical analysis of enamel and dentin following the application of three different at-home bleaching systems. Oper. Dent..

[21] Inonu E., Hakki S.S., Kayis S.A., Nielsen F.H. (2020). The Association Between Some Macro and Trace Elements in Saliva and Periodontal Status. Biol. Trace Elem. Res..

[22] Sánchez A.R., Rogers R.S., Sheridan P.J. (2004). Tetracycline and other tetracycline-derivative staining of the teeth and oral cavity. Int. J. Dermatol..

[23] Soares D.G., Marcomini N., Basso F.G., Pansani T.N., Hebling J., de Souza Costa C.A. (2016). Influence of Restoration Type on the Cytotoxicity of a 35% Hydrogen Peroxide Bleaching Gel. Oper. Dent..

[24] Soares D.G., Gonçalves Basso F., Hebling J., de Souza Costa C.A. (2015). Effect of hydrogen-peroxide-mediated oxidative stress on human dental pulp cells. J. Dent..

